# Comparison of clinicopathological features and prognostic significance between synchronous multiple primary and solitary esophageal squamous cell carcinomas

**DOI:** 10.1186/s12885-022-10283-2

**Published:** 2022-11-19

**Authors:** Yueyang Yang, Peng Tang, Mingquan Ma, Hongdian Zhang, Haitong Wang, Kai Zhu, Wanyi Xiao, Lei Gong, Zhentao Yu

**Affiliations:** 1 Department of Esophageal Cancer, Tianjin Medical University Cancer Institute and Hospital, National Clinical Research Center for Cancer, Key Laboratory of Cancer Prevention and Therapy, Tianjin’s Clinical Research Center for Cancer, Tianjin, China; 2 National Cancer Center/National Clinical Research Center for Cancer/Cancer Hospital & Shenzhen Hospital, Chinese Academy of Medical Sciences, PeKing Union Medical College, Shenzhen, China

**Keywords:** Esophageal squamous cell carcinoma, Multiple primary cancer, Lymph node metastasis, Prognosis

## Abstract

**Background:**

Synchronous multiple primary esophageal squamous cell carcinoma (S-MPESCC) refers to more than one primary esophageal carcinoma detected in a solitary patient at the time of initial presentation. The purpose of this study was to evaluate the clinicopathological features, appropriate surgical approaches and long-term survival in patients with S-MPESCC by comparing with those with solitary esophageal squamous cell carcinoma (SESCC).

**Methods:**

In total, 567 patients with esophageal squamous cell carcinoma surgically resected in Tianjin Medical University Cancer Institute and Hospital from January 2012 to December 2018 were screened for retrospective analysis (50 in the S-MPESCC group and 516 in the SESCC group).

**Results:**

No significant difference was observed in terms of other characteristics except total alcohol consumption (*P* = 0.029). S-MPESCC had higher lymph node rate than SESCC (62.0% and 44.1%, respectively; *P* = 0.015) especially in upper mediastinal (32.0% and 18.6%, respectively; *P* = 0.023) and abdominal (38.0% and 22.8%, respectively; *P* = 0.017) regions. The survival was not different between the two groups, and the 5-year survival rates of S-MPESCC and SESCC were 46.2% and 50.8%, respectively (*P* = 0.507). But for patients with pT3-4 cancers, the survival in S-MPESCC was worse than that in SESCC (*P* = 0.033). In the multivariate analysis, pT stage of primary cancer was an important independent predictor of prognosis in patients with S-MPESCC (hazard ratio [HR], 3.968; 95% confidence interval [CI], 1.031 to 15.268; *P* = 0.045).

**Conclusions:**

S-MPESCC was significantly different from SESCC in terms of clinicopathological characteristics include alcohol intake and pattern of lymphatic metastasis. Furthermore, S-MPESCC showed worse long-term survival than SESCC with increasing depth of primary cancer infiltration.

**Supplementary Information:**

The online version contains supplementary material available at 10.1186/s12885-022-10283-2.

## Background

Multiple primary carcinoma (MPC), first reported by Billroth in 1889 and standardized by Warren and Gates in 1932, is defined as two or more primary carcinomas pathologically confirmed in different parts of an organ or in different organs synchronously (within 6 months) or metachronously (more than 6 months) [1]. Most MPC with esophageal cancer (MPWEC) occurred in stomach, head-and-neck, and lungs with an incidence from 9.5% to 21.9% [2, 3]. Reports on synchronous MPWEC (SMPWEC) have increased due to prolonged lifespan and improvements in diagnostic techniques. But it has been proved a poor prognosis due to heavy tumor burdens and complex treatment strategies [4]. What is more, the mechanism of SMPWEC is still controversial. Most studies have adopted the concept of “field cancerization”, first mentioned by Slaughter in 1953 [5], which exposed the epithelium from the head and neck, esophagus, or lung to common carcinogenic agents such as tobacco smoke and alcoholism lead to multiple carcinomas [6, 7].

Synchronous multiple cancers in the same organ are well known in colorectal, with the incidence range from 1.1% to 8.1% [8]. But they are rarely reported in esophagus. Synchronous multiple primary esophageal squamous cell carcinoma (S-MPESCC) was characterized by the presence of more than one primary separate tumors on the esophagus derived from the same genetic and environmental background, which detected in one solitary patient by the time of initial diagnosis with the incidence rates at about 1.0%–3.42% [1, 9]. Previous studies on SMPWEC have focused on synchronous primary esophageal cancer with tumor on the other organs, such as head and neck, gastrointestinal tract, or lungs [3, 7]. However, fewer studies have been focused on S-MPESCC at different locations in one esophagus, which resulted in lack of consensus on its clinicopathological features, surgical approaches, and prognosis.

It was a retrospectively study comparing the clinical futures and prognosis between S-MPESCC and solitary esophageal squamous cell carcinoma (SESCC) under surgical treatment in our hospital. And we further explored the pattern of the lymph node metastasis (LNM) and the risk factors on survival in patients with S-MPESCC. We hope that it could provide some guidance for future surgical treatment of the S-MPESCC.

## Methods

### Study population

**Fig. 1:**
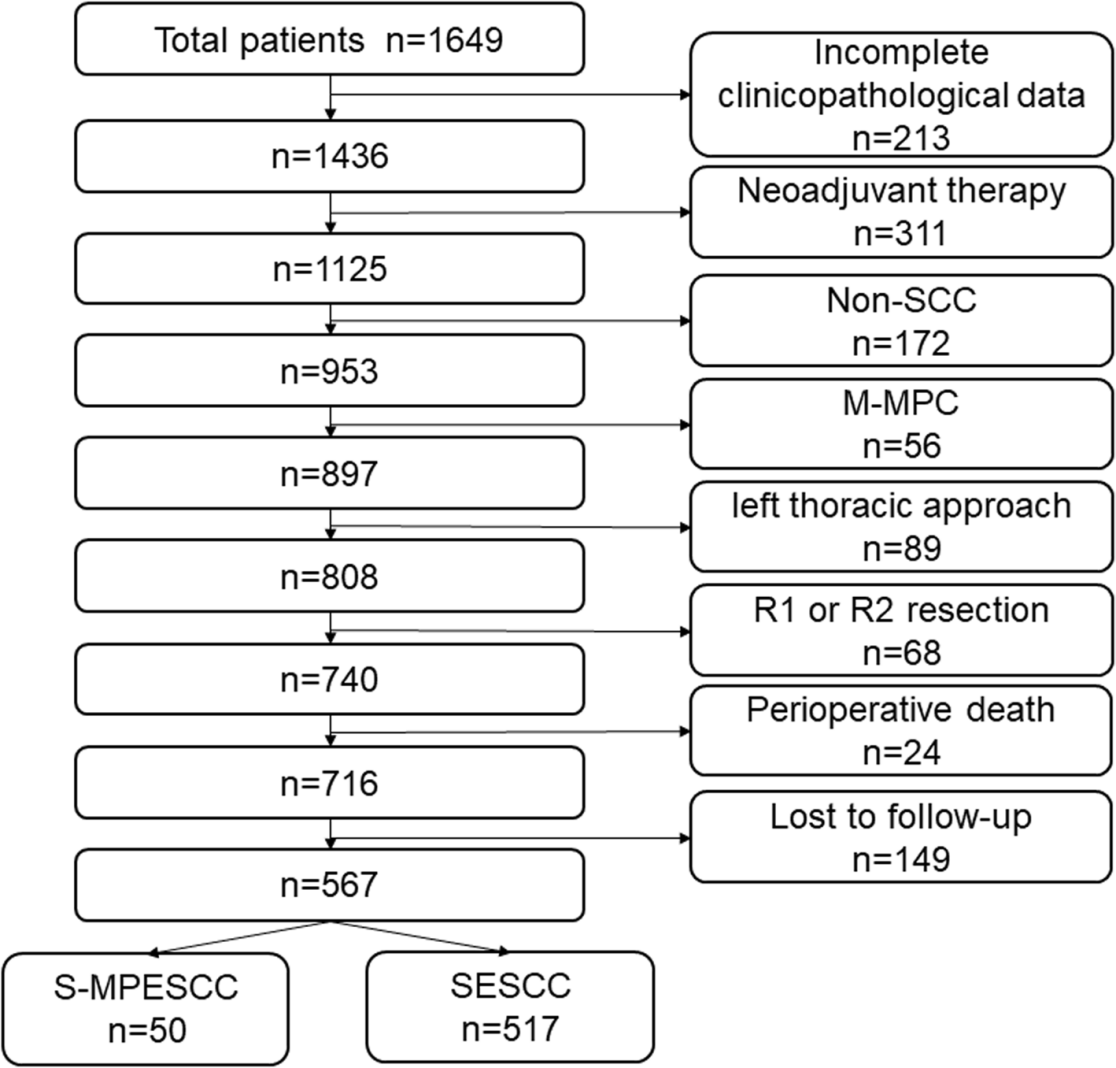
Flowchart summarizing study selection

All patients with esophageal cancer who underwent surgery between January 2012 and December 2018 were reviewed. The flowchart of patient enrollment was shown in Fig. 1. A total of 567 patients were finally included into our study for analysis by using the following inclusion criteria: (i) pathologically confirmed thoracic esophageal squamous cell carcinoma, (ii) surgical methods of right thoracic approach, (iii) no patient with imperfect clinical data. And patients were excluded using the following exclusion criteria: (i) with distant metastasis confirmed using imaging, (ii) receiving neoadjuvant radiotherapy and chemotherapy, (iii) with palliative resection, (iv) with preoperative or postoperative occurrence of primary malignant tumors in other organs. The study was approved by the Institutional Ethics Committee of the Tianjin Medical University Cancer Institute and Hospital (No. bc2022093), and the need for informed consent was waived by the ethics committee/Institutional Review Board of the Tianjin Medical University Cancer Institute and Hospital. All methods were carried out in accordance with relevant guidelines and regulations.

Total alcohol consumption was expressed in grams of ethanol calculated by using a standard conversion formula for Chinese spirits. Wine was assumed to be 40–60% ethanol (v/v). Brinkman Index, which was calculated by daily consumption times years of smoking, was evaluated to define the amount of cigarette smoking.

### Definition of S-MPESCC

On the basis of the criteria proposed by Warren and Gates in 1932, MPC was usually diagnosed in accordance with the following principles [1]. Each lesion must be (i) pathologically malignant, (ii) separated by the normal mucosa, and (iii) not the result of local extension or metastasis of another lesion confirmed by pathology examination after esophagectomy. The definition of S-MPESCC was also combined with the criteria proposed by Moertel [10]. (i) Each lesion must be pathologically identified as malignant or tis. (ii) Cancer lesion with the deepest invasion or the longest length if occurring in the same pT stage should be defined as primary cancer, and other cancer sites should be defined as multiple cancer lesions. Thus, all patients were divided into two groups, i.e., S-MPESCC (50 patients) and SESCC (517 patients) groups. And to distinguish the name of different cancer foci, we named the locations of primary cancer by capital letter, such as U (Upper esophagus), M (Middle esophagus) and L (Lower esophagus). Likewise, the locations of multiple cancer were named by lower case letter, such as u (upper esophagus), m (middle esophagus) and l (lower esophagus). Therefore, these 50 patients with S-MPESCC were divided into eight groups which was called as U-m, U-l, M-u, M-m, M-l, L-u, L-m and L-l, respectively (Supplementary Table 1). We also listed the infiltration depth of each lesionin Supplementary Table 2.

### Surgical procedure and pathology classification

All target cases were operated on using the right thoracic approach, including the Ivor–Lewis and McKeown methods, after a comprehensive examination. According to the 8th AJCC cancer staging system, cervical lymph nodes (LNs) were named as 1L/R, dissected thoracic LNs were named as 2L/R, 8U, 4L/R, 7, 8 M, 8Lo, and 9R nodes, and abdominal LNs were named as 16, 17, 18, and 19 nodes. The location of thoracic esophageal carcinoma and the pathologic TNM stage of disease were also determined using the same system. The lymph node rate (LNR) was defined as the ratio of the cases of patients occurring lymph node metastasis to total cases.

### Follow-up

The survival status of each patient was followed up using a combination of outpatient records and phone calls as of December 2021. The follow-up period ranged from 1 to 118 months, and the median follow-up time was 39 months. Overall survival (OS) was calculated from the date of the surgery to the occurrence of the event or to the last known date of the follow-up.

### Statistical analysis

The cutoff of relevant variables was determined using the receiver operating characteristic (ROC) curve (Supplementary Fig. 1). Thus, the length of the primary tumor was divided into < 3.75 and ≥ 3.75 cm groups (cutoff = 3.75), and the number of LNs dissection was divided into < 24 and ≥ 24 groups (cutoff = 23.5). The binary logistic regression analysis was used to detect the independent factors of lymph nodes metastasis. The Kaplan–Meier method was used to draw the survival curve. The log-rank method was used for survival analysis, and the Cox regression model was used to determine independent prognostic factors. Statistical analyses were performed using the SPSS 25.0 statistical software, and probability (*P*) values ≤ 0.05 were considered statistically significant.

## Results

### Comparison of the characteristics of S-MPESCC and SESCC groups

Of all the 567 patients enrolled in this study, 50 (8.8%) had S-MPESCC and 517 (91.2%) had SESCC. The comparison of the clinicopathological characteristics of patients in S-MPESCC and SESCC groups was summarized in Table 1. All general clinicopathological features were similar between these two groups except for total alcohol consumption (*P* = 0.029). No significant differences were observed in other variables, such as age, smoking, location of primary tumor, operation approaches, length of primary tumor, pT stage of primary tumor, pTNM stage of primary tumor and the number of dissected LNs. But a greater proportion of positive LNs was observed in the S-MPESCC group than the SESCC group (62.0% vs. 44.1%; *P* = 0.015). Risk factors associated with LNM across the enrolled patients were summarized in Table 2. It indicated that S-MPESCC was an independent prognostic factor affecting LNM ( *P* = 0.010), which suggested that S-MPESCC was more prone to occur LNM than SESCC.

**Table 1:** The comparison of the clinicopathological characteristics of patients in S-MPESCC and SESCC groups

Variables	S-MPESCC	SESCC	*P*
	(*n* = 50)	(*n* = 517)	
Age, n (%)			0.906
< 60	22(44.0%)	223(43.1%)	
≥ 60	28(56.0%)	294(56.9%)	
Smoking, n (%)			0.520
No	13(26.0%)	157(30.4%)	
Yes	37(74.0%)	360(69.6%)	
Tobacco (Brinkman index)	400	400	0.984
Drinking Alcohol, n (%)			0.144
No	14(28.0%)	199(38.5%)	
Yes	36(72.0%)	318(61.5%)	
Total alcohol consumption, drinks	416.7	250	0.029
Location of primary cancer, n (%)			0.346
Upper	3(6.0%)	60(11.6%)	
Middle	32(64.0%)	336(65.0%)	
Lower	15(30.0%)	121(23.4%)	
Operation, n (%)			0.249
Ivor-Lewis	8(16.0%)	55(10.6%)	
Mckeown	42(84.0%)	462(89.4%)	
Length of primary cancer, n (%)			0.829
< 3.75 cm	29(58.0%)	308(59.6%)	
≥ 3.75 cm	21(42.0%)	209(40.4%)	
pT stage of primary cancer, n (%)			0.701
T1	12(24.0%)	90(17.4%)	
T2	9(18.0%)	111(21.5%)	
T3	15(30.0%)	160(30.9%)	
T4	14(28.0%)	156(30.2%)	
pTNM stage of primary cancer, n (%)			0.071
I	10(20.0%)	72(13.9%)	
II	8(16.0%)	163(31.5)	
III	29(58.0%)	263(50.9%)	
IV	3(6.0%)	19(3.7%)	
LNM, n (%)			0.015
Absent	19(38.0%)	289(55.9%)	
Present	31(62.0%)	228(44.1%)	
pN stage, n (%)			0.049
N0	19(38.0%)	289(55.9%)	
N1	16(32.0%)	138(26.7%)	
N2	12(24.0%)	71(13.7%)	
N3	3(6.0%)	19(3.7%)	
Dissection number of lymph nodes, n (%)			0.899
< 24	27(54.0%)	284(54.9%)	
≥ 24	23(46.0%)	233(45.1%)	

*LNM* lymph node metastasis*, S-MPESCC* synchronous multiple primary esophageal squamous cell carcinoma*, SESCC* single esophageal squamous cell carcinoma*, 1 Drink* = *12 g ethanol*

**Table 2:** The univariate and multivariate analyses of factors associated with LN metastasis for all patients

Variables	LNM		*P* (univariate)	*P* (multivariate)
	No(*n* = 308)	Yes(*n* = 259)		
Age, n (%)			0.300	-
< 60	127(41.2%)	118(45.6%)		
≥ 60	181(58.8%)	141(54.4%)		
Smoking, n (%)				
No	99(32.1%)	71(27.4%)	0.221	
Yes	209(67.9%)	188(72.6%)		
Tobacco (Brinkman index)	400	400	0.243	
Drinking Alcohol, n (%)			0.273	-
No	122(39.6%)	91(35.1%)		
Yes	186(60.4%)	168(64.9%)		
Total alcohol consumption, drinks	250	250	0.119	-
Location of primary cancer, n (%)			0.038	0.088
Upper	37(12.0%)	26(10.0%)		
Middle	210(68.2%)	158(61.0%)		
Lower	61(19.8%)	75(29.0%)		
Operation, n (%)			0.743	-
Ivor-Lewis	33(10.7%)	30(11.6%)		
Mckeown	275(89.3%)	229(88.4%)		
Length of primary cancer, n (%)			0.088	0.547
< 3.75 cm	193(62.7%)	144(55.6%)		
≥ 3.75 cm	115(37.3%)	115(44.4%)		
pT stage of primary cancer, n (%)			< 0.001	< 0.001
T1	80(26.0%)	22(8.5%)		
T2	71(23.1%)	49(18.9%)		
T3	85(27.6%)	90(34.7%)		
T4	72(23.4%)	98(37.8%)		
Dissection number of lymph nodes, n (%)			< 0.001	0.002
< 24	191(62.0%)	120(46.3%)		
≥ 24	117(38.0%)	139(53.7%)		
Cohort, n (%)			0.015	0.010
SESCC	289(93.8%)	228(88.0%)		
S-MPESCC	19(6.2%)	31(12.0%)		

*LNM* lymph node metastasis*, S-MPESCC* synchronous multiple primary esophageal squamous cell carcinoma*, SESCC* single esophageal squamous cell carcinoma*, 1 Drink* = *12 g ethanol*

### Characteristics of the S-MPESCC group

**Fig. 2:**
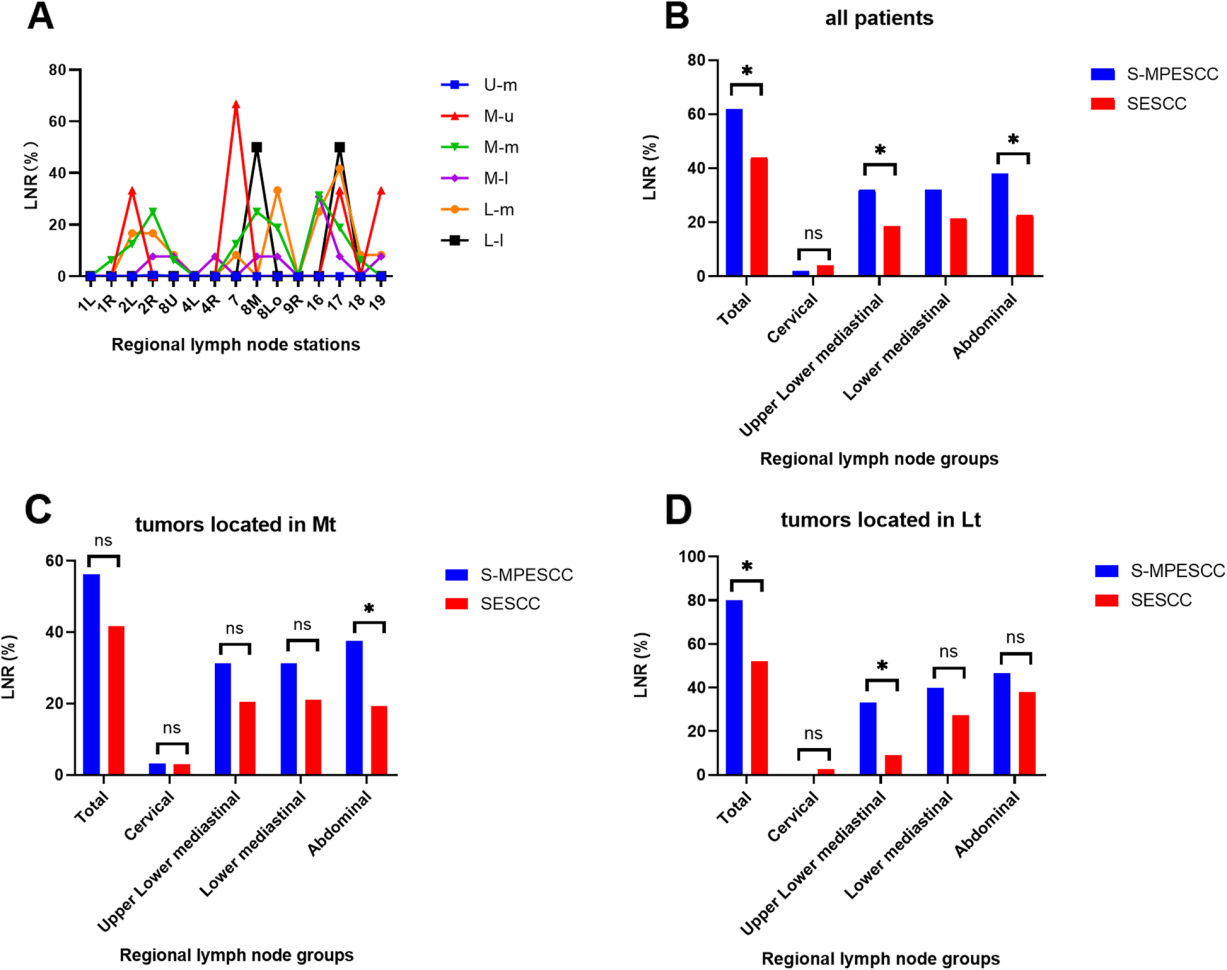
**A** LNR at LN stations in the S-MPESCC group according to different tumor location groups. (B-D) Comparison of LNR at different LN regions between the S-MPESCC and SESCC groups: **B** for all patients; for tumor located in **C **Mt and **D **Lt. *LNR lymph node ratio, LN lymph node, Mt middle thoracic esophagus, Lt lower thoracic esophagus, S-MPESCC synchronous multiple primary esophageal squamous cell carcinoma, SESCC single esophageal squamous cell carcinoma*

For the S-MPESCC group, there were a total of 44 patients had dual tumors and 6 had triple tumors. From the results of pathology outcomes, it showed that multiple sites had a relatively close distance with each tumor site. M-m, M-l and L-m had the highest incidence rates at 32.0%,26.0% and 24.0%, respectively. The incidence of L-l (4.0%) was relatively low. There was just one patient (2%) in both the U-l and L-u groups, which had no LNM. Thus, U-l and L-u groups were excluded from the following comparative analyses. The LNR at each station was displayed in Fig. 2A. The LNM was more prone to occur in the 2L/R, 8U, 7, 8 m, 8L, 16, and 17 stations regardless the location of the primary and multiple cancer. Taken together, the above LN stations were selected for the following comparative analyses.

### Comparison of metastatic patterns of LN between S-MPESCC and SESCC groups

Of 50 patients collected in the S-MPESCC group, there were only three patients with primary cancer located at the upper esophagus, which only one case was found the presence of LNM at recurrent laryngeal region. Then we excluded this subgroup from the following comparative analyses. In return, we collected the LNR at different LN regions and LN stations in Table 3 and Fig. 2B-D. And we also made a subgroup analysis, which showed the comparison of LNR for patients whose primary tumor located at the middle and lower esophagus between the S-MPESCC and SESCC groups. Compared with SESCC, patients with S-MPESCC were observed to have a higher proportion of LNM in the upper mediastinal (32.0% vs. 18.6%; *P* = 0.023) and abdominal regions (38.0% vs. 22.8%; *P* = 0.017). For the subgroup with primary cancer located in middle esophagus, the LNR in the S-MPESCC group was significantly higher than the SESCC group in the abdominal regions (37.5% vs. 19.3%; *P* = 0.016) especially at station 16 (28.1% vs. 12.5%; *P* = 0.015). For the subgroup with primary cancer located in lower esophagus, LNM was found more frequent in upper mediastinal regions in S-MPESCC group than the SESCC group (33.3% vs. 9.1%; *P* = 0.017), especially at station 2L/R (26.7% vs. 7.4%; *P* = 0.038).

**Table 3:** Comparison of LNR at different LN regions and LN stations between the S-MPESCC and SESCC groups

LN regions and stations		All esophageal cancer			Mt esophageal cancer			Lt esophageal cancer		
		S-MPESCC (*n* = 50)	SESCC (*n* = 517)	*P*	S-MPESCC (*n* = 32)	SESCC (*n* = 336)	*P*	S-MPESCC (*n* = 15)	SESCC (*n* = 121)	*P*
Total, n (%)		31 (62.0%)	228 (44.1%)	0.015	18 (56.3%)	140 (41.7%)	0.111	12 (80.0%)	63 (52.1%)	0.040
Cervical, n (%)	Subtotal	1 (2.0%)	21 (4.1%)	0.711	1 (3.1%)	10 (3.0%)	1.000	0 (0)	3 (2.5%)	1.000
Upper Mediastinal, n (%)	2L/R	13 (26.0%)	78 (15.1%)	0.045	8 (25.0%)	57 (17.0%)	0.255	4 (26.7%)	9 (7.4%)	0.038
	8U	3 (6.0%)	21 (4.1%)	0.460	2 (6.3%)	17 (5.1%)	0.676	1 (6.7%)	1 (0.8%)	0.209
	Subtotal	16 (32.0%)	96 (18.6%)	0.023	10 (31.3%)	69 (20.5%)	0.158	5 (33.3%)	11 (9.1%)	0.017
Lower Mediastinal, n (%)	7	8 (16.0%)	52 (10.1%)	0.192	7 (21.9%)	39 (11.6%)	0.098	1 (6.7%)	11 (9.1%)	1.000
	8 M	6 (12.0%)	45 (8.7%)	0.437	5 (15.6%)	30 (8.9%)	0.210	1 (6.7%)	12 (9.9%)	1.000
	8Lo	8 (16.0%)	44 (8.5%)	0.080	4 (12.5%)	27 (8.0%)	0.330	4 (26.7%)	14 (11.6%)	0.114
	Subtotal	16 (32.0%)	111 (21.5%)	0.088	10 (31.3%)	71 (21.1%)	0.187	6 (40.0%)	33 (27.3%)	0.365
Abdominal, n (%)	16	12 (24.0%)	77 (14.9%)	0.091	9 (28.1%)	42 (12.5%)	0.015	3 (20.0%)	33 (27.3%)	0.759
	17	11 (22.0%)	68 (13.2%)	0.085	5 (15.6%)	36 (10.7%)	0.380	6 (40.0%)	26 (21.5%)	0.119
	Subtotal	19 (38.0%)	118 (22.8%)	0.017	12 (37.5%)	65 (19.3%)	0.016	7 (46.7%)	46 (38.0%)	0.517

*LNR* lymph node ratio*, LN* lymph node*, Mt* middle thoracic esophagus*, Lt* lower thoracic esophagus*, S-MPESCC* synchronous multiple primary esophageal squamous cell carcinoma*, SESCC* single esophageal squamous cell carcinoma

### Risk factors of LNM in the S-MPESCC group

Factors influencing LNM in patients with S-MPESCC were presented in Table 4. The result of logistic analysis revealed that deeper invasion of primary (*P* < 0.001) and multiple cancers (*P* = 0.042) were risk factors for LNM in patients with S-MPESCC. We then incorporated the variables with p-values less than or close to 0.1 in the binary logistic regression analysis into the multivariate logistic proportional hazard models. These variables included length of primary cancer, pT stage of primary cancer, pT stage of multiple cancer and dissection number of lymph nodes. The results of multivariate analysis showed that the depth of primary cancer invasion was an independent prognostic factor for LNM (*P* = 0.023). In addition, the number of dissected LNs ≥ 24 also had independent significance for the detection of positive LNs (*P* = 0.038).

**Table 4:** The univariate and multivariate analyses of factors associated with LN metastasis in the S-MPESCC group

Variables	LNM		*P* (univariate)	*P* (multivariate)
	No(*n* = 19)	Yes(*n* = 31)		
Age, n (%)			0.833	-
< 60	8(42.1%)	14(45.2%)		
≥ 60	11(57.9%)	17(54.8%)		
Smoking, n (%)				
No	4(21.1%)	9(29.0%)	0.532	
Yes	15(78.9%)	22(71.0%)		
Tobacco (Brinkman index)	400	300	0.504	
Drinking Alcohol, n (%)			0.238	-
No	3(15.8%)	11(35.5%)		
Yes	16(84.2%)	20(64.5%)		
Total alcohol consumption, drinks	500	375	0.162	-
Location of primary cancer, n (%)			0.156	-
Upper	2(10.5%)	1(3.2%)		
Middle	14(73.7%)	18(58.1%)		
Lower	3(15.8)	12(38.7%)		
Operation, n (%)			1.498	
Ivor-Lewis	1(5.3%)	7(22.6%)		-
Mckeown	18(94.7%)	24(77.4%)		
Length of primary cancer, n (%)			0.079	0.220
< 3.75 cm	14(73.7%)	15(48.4%)		
≥ 3.75 cm	5(26.3%)	16(51.6%)		
pT stage of primary cancer, n (%)			< 0.001	0.023
T1	10(52.6%)	2(6.5%)		
T2	5(26.3%)	4(12.9%)		
T3	2(10.5%)	13(41.9%)		
T4	2(10.5%)	12(38.7%)		
pT stage of multiple cancer, n (%)			0.042	0.138
T1-4	11(57.9%)	26(83.9%)		
Tis	8(42.1%)	5(16.1%)		
Dissection number of lymph nodes, n (%)			0.109	0.038
< 24	13(68.4%)	14(45.2%)		
≥ 24	6(31.6%)	17(54.8%)		

*LN* lymph node*, LNM* lymph node metastasis*, S-MPESCC* synchronous multiple primary esophageal squamous cell carcinoma*, Tis* tumor in situ*, 1 Drink* = *12 g ethanol*

### Difference of prognosis between the S-MPESCC and SESCC groups

**Fig. 3:**
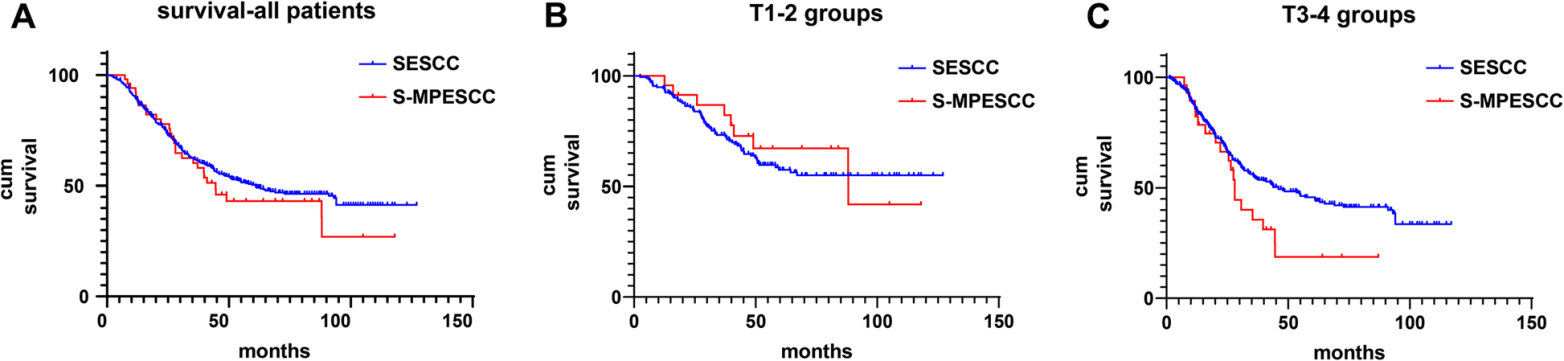
**A**-**C** Kaplan–Meier survival curves between the S-MPESCC and SESCC groups: **A** for all patients; **B** for T1–2 group; **C** for T3–4 group

The outcomes of the prognostic analysis were shown in Fig. 3. In the entire cohort, there was no significant difference in 5-year OS between the S-MPESCC and SESCC groups (Fig. 3A; 46.2% vs 50.8%; *P* = 0.507). Kaplan–Meier curves for OS according to pT stage according to the primary cancer are shown in Figs. 3B and C, respectively. No statistically difference in 5-year OS was observed between the two groups at stage pT1-2 subgroup (Fig. 3B). However, patients in S-MPESCC group tended to have a poorer survival than those in SPESCC group at stage pT3-4 subgroup (Fig. 3C; 19.7% vs 46.9%; *P* = 0.033). We then performed a Cox multivariate analysis of patients at stage pT3-4, it also revealed that S-MPESCC (*P* = 0.047) was an independent risk factor (Table 5). For patients in S-MPESCC group, the results of the univariate and multivariate analyses for OS were shown in Table 6. The results of the univariate analyses revealed that significant difference in OS was observed in some variables, such as smoking (*P* = 0.020), Brinkman index (*P* = 0.008), location of primary cancer (*P* = 0.045), pT stage of primary cancer (*P* = 0.001), pT stage of multiple cancer (*P* = 0.018) and pN stage (*P* = 0.028). The results from the multivariate Cox regression analysis indicated a worse survival for patients with smoking habit (hazard ratio [HR], 8.561; 95% **c**onfidence interval [CI], 1.475 to 49.704; P = 0.017) and patients at stage pT3-4 of primary cancer (hazard ratio [HR], 3.968; 95% confidence interval [CI], 1.031 to 15.268; *P* = 0.045).

**Table 5:** The univariate and multivariate analyses on prognostic factors in T3–4 group

Variables	Univariate analysis		Multivariate analysis	
	Cases	*P*	HR (95%CI)	*P*
Age, n (%)		0.851	-	-
< 60	144(41.7%)			
≥ 60	201(58.3%)			
Smoking, n (%)		0.237		
No	100(29.0%)			
Yes	245(71.0%)			
Tobacco (Brinkman index)		0.065		0.285
Drinking Alcohol, n (%)		0.116	-	-
No	133(38.6%)			
Yes	212(61.4%)			
Total alcohol consumption	-	0.023	-	0.078
Location of primary cancer, n (%)		0.029		0.065
Upper	38(11.0%)		Ref	
Middle	220(63.8%)		2.021(1.110–3.678)	0.021
Lower	87(25.2%)		1.923(1.003–3.684)	0.049
Operation, n (%)		0.474	-	-
Ivor-Lewis	47(13.6%)			
Mckeown	298(86.4%)			
Length of primary cancer, n (%)		0.342	-	-
< 3.75 cm	162(47.0%)			
≥ 3.75 cm	183(53.0%)			
Dissection number of lymph nodes, n (%)		0.062		0.030
< 24	174(50.4%)		Ref	
≥ 24	171(49.6%)		0.729(0.536–0.991)	
Cohort, n (%)		0.033		0.047
SESCC	316(91.6%)		Ref	
S-MPESCC	29(8.4%)		1.677(1.022–2.752)	

*S-MPESCC* synchronous multiple primary esophageal squamous cell carcinoma*, SESCC* single esophageal squamous cell carcinoma

**Table 6:** The univariate and multivariate analyses on prognostic factors in the S-MPESCC group

Variable	Univariate analysis	Multivariate analysis	
	*P*	HR (95%CI)	*P*
Age	0.327	-	-
< 60			
≥ 60			
Smoking	0.020		0.017
No		Ref	
Yes		8.561 (1.475–49.704)	
Tobacco (Brinkman index)	0.008		0.610
Drinking Alcohol	0.137	-	-
No			
Yes			
Total alcohol consumption	0.307	-	-
Location of primary cancer	0.045		0.521
Upper		Ref	
Middle		1.008 (0.102–9.944)	0.995
Lower		1.695(0.148–19.476)	0.672
Operation	0.286	-	-
Ivor-Lewis			
Mckeown			
Length of primary cancer	0.205	-	-
< 3.75 cm			
≥ 3.75 cm			
pT stage of primary cancer	0.001		0.045
T1-2		Ref	
T3-4		3.968(1.031–15.268)	
pT stage of multiple cancer	0.018		0.153
T1-4		Ref	
Tis		0.293(0.054–1.580)	
pN stage	0.028		0.906
N-		Ref	
N +		1.081(0.295–3.964)	
Dissection number of lymph nodes	0.137	-	-
< 24			
≥ 24			

*S-MPESCC* synchronous multiple primary esophageal squamous cell carcinoma, *Tis* tumor in situ

## Discussion

Esophageal cancer (EC) is one of the most common gastrointestinal malignancies in the world. In China, 90% of the pathological types of EC patients are squamous cell carcinoma. Currently, the clinical features, diagnosis and treatment of SESCC are well understood [11]. However, the clinicopathological characteristics of S-MPESCC are not well defined. And there are also no standardized guidelines of the treatment of S-MPESCC, which is due to the relatively low incidence at around 1.0%–3.42% [1, 9].

Multiple carcinogenesis occurred in one organ can be explained by “field carcinogenesis theory”. It postulates that different areas of tissue simultaneously acquired genetic instability and variability within one original clone due to prolonged exposure to carcinogens, which resulted in the evolutionary process in tumorigenesis [12]. As a hollow organ for transporting food, the entire esophageal epithelial surface is exposed to the same carcinogenic insults. According to the theory, when one mucosal epithelial cell turns cancerous under the continued presence of carcinogen, other multiple epithelial precancerous lesions are also prone to cancerization due to the same carcinogen, resulting in the formation of multiple primary esophageal cancer [13]. As one of the representative examples of field carcinogenesis, the incidence of S-MPESCC is quite high in heavy smokers and heavy drinkers [14–16]. As the most common carcinogen, alcohol drinking can induce genetic/epigenetic alterations in esophageal mucosal cells. Morita et al. [17] proposed that excessive exposure to environmental carcinogens, such as alcohol, may cause a FHIT loss in the esophageal epithelium. And a loss of FHIT expression was also associated with multicentric carcinogenesis. In addition, p53 alterations were the other key molecular events in multifocal carcinogenesis in the esophagus, especially in alcoholics with inactive aldehyde dehydrogenase-2 (ALDH2) [18].

In the present study, we found that more than 416.7 drinks of total alcohol consumption promoted the occurrence of S-MPESCC by comparing with the SESCC group. Considering the “field cancerization theory”, epithelial precancerous lesions or cancer lesions can occur multicentrically in one same esophagus. Therefore, accurate preoperative evaluation for patients with multiple cancers in the esophagus seems to be very important, especially for those who had total alcohol intake more than 416.7 drinks. Lu’s iodine staining can help detect esophageal mucosal lesions [19, 20]. However, some studies have indicated that the Narrow Band Imaging (NBI) endoscopy was superior to the Lugol chromoendoscopy in detecting multiple lesions [21, 22]. The sensitivity of 18F-FDG-PET/CT in the diagnosis of second primary cancer could reach to 95.24% [23]. Thus, the combination of 18F-FDG-PET/CT and esophagoscopy may be a good choice for the diagnosis of S-MPESCC.

Consistent with SESCC, the location of primary cancer in S-MPESCC was also higher in the middle thoracic esophagus [24]. As mentioned in our study, M-m, M-l and L-m shared the highest incidence at the rates of 32.0%,26.0% and 24.0%, respectively, which reflected that most multiple lesions of the S-MPESCC were close to each other. The incidence of L-l (4.0%) was relatively low, which may be related to the high incidence of synchronous esophageal cancer with esophagogastric junction and gastric cancers [4, 25]. Therefore, we advocated that when major primary cancer foci are found in the middle thoracic esophagus, more attention should be paid to the surrounding mucosa to detect other concomitant tumor. In addition, U-l and L-u still occurred in 4% of patients. Based on these findings, we suggested that all patients with S-MPESCC should receive McKeown approaches to avoid the tumor residue. However, complete surgical resection is difficult when multiple cancer sites located at the cervical esophagus. If the multiple lesions located at the cervical esophagus are precancerous lesions or carcinoma in situ, a staging resection procedure by endoscopic submucosal dissection (ESD) and esophagectomy may be a new option [26, 27].

In recent years, although the multidisciplinary methods have achieved great advances, surgical resection plus systematic LN dissection remains the standard treatment for potentially resectable esophageal cancer [28, 29]. A recognition of the lymphatic drainage system of the esophagus based on the anatomical mesentery is crucial to understanding the LN dissection strategy. By an anatomical study of serial transverse thin sections, Kuge et al. indicated that there were two primary lymphatic drainage system. One was the very evident long longitudinal lymphatic extension in the esophageal submucosa mainly drain directly to their proximal and distal ends, the other was the lymphatic routes to paraoesophageal lymph nodes usually originate from the intermuscular area of the muscularis propria [30]. It indicated that the location of positive LNs did not reflect the anatomical distance from the primary tumor due to the lymphatic drainage system presented above [31–33]. Specifically, the upper thoracic tumors had a higher metastatic frequency of upper mediastinal nodes. Patients with tumor in the middle esophagus also tended to involve upper mediastinal area. In patients with tumor in the lower middle esophagus, abdominal LNs had the highest incidence of metastasis. Therefore, the extent of dissection should be determined according to the incidence of LNM rather than to the anatomical distance from the tumor.

As shown in our results, the LNR in patients with S-MPESCC was 62.0%, whereas that of patients with SESCC was 44.1% (*P* = 0.015). For the subgroup with primary cancer located in middle esophagus, patients with S-MPESCC had the higher incidence of metastasis in the abdominal regions than SESCC (37.5% vs. 19.3%; *P* = 0.016). For the subgroup with primary cancer located in lower esophagus, LNM was found more frequent in upper mediastinal regions in patients with S-MPESCC than SESCC (33.3% vs. 9.1%; *P* = 0.017). It indicated that S-MPESCC showed a unique metastasis trend which was different from SESCC. But this phenomenon is not difficult to explain based on the lymph node drainage system described above. When the primary cancer was located in the middle thoracic esophagus, the other multiple cancer foci often occurred in the middle and lower esophagus, which resulted in the high incidence of LNM in abdominal region. Similarly, for patients with primary cancer located in the lower thoracic esophagus, the other multiple cancer foci often occurred in the middle esophagus, which resulted in the high incidence LNM in the upper mediastinal region. We can see that multiple cancer sites in the S-MPESCC group played a key role for the preferred areas of LNM. Thus, extent of LNs dissection should be considered for patients with S-MPESCC according to the location of esophageal multiple cancer.

Several studies have proved that the metastasis rate of regional lymph nodes was positively related with the depth of tumor invasion, which was consistent with the anatomical basis of the lymph node drainage system described above [34–36]. For surgical treatment, adequate harvested number of LNs is essential for ensuring the staging accuracy. However, attempts to identify a minimum lymphadenectomy to optimize survival and tumor staging have not been reached a uniform standard, with recommendations of LNs dissection ranging widely from 6 to 40 nodes [37, 38]. The National Comprehensive Cancer Network (NCCN) has proposed that at least 15 LNs should be removed to sufficiently evaluate for nodes metastases [39]. In this study, the status of pT stage of primary cancer was an independent risk factor for LNM in the S-MPESCC group. In addition, more than 24 harvest LNs were an independent risk factor for the detection of positive LNs in patients with S-MPESCC.

One previous study included 52 cases of unresectable synchronous multiple primary esophageal cancer has found that the 1-, 3-, and 5-year survival rates and median survival time were 65.4%, 17.3%, 7.7%, and 15.0 months, respectively [9]. And the multivariate survival analysis showed that tumor length and distant metastasis were independent prognostic factors. In contrast to that research, the current study enrolled patients with resectable S-MPESCC who have a lower tumor burden and a lower risk of metastasis. As a result, the 3- and 5-year cumulative survival rates were 62.0%, and 46.2%, respectively. Prior study has shown that the OS rates at 3 and 5 years for patients with SESCC who receiving surgery alone were 57.8% and 49.1%, respectively [40]. It shows that the OS was no significant difference between S-MPESCC and SESCC, which is consistent with our findings. But in the pT3–4 subgroup, the OS of S-MPESCC was significantly worse compared with the SESCC group. Several reasons might have contributed to this result. First, we find that S-MPESCC was more prone to occur LNM than SESCC. The strongest risk factors, which were associated with positive LNs, reflected cancer growth, biology, histology, and prognosis [41, 42]. Second, the increasing depth of primary cancer invasion resulted in increasing numbers of LNM, which caused a worse prognosis for S-MPESCC patients. All these finds were also demonstrated in other studies, that late pT stage was strongly associated with LNM and prognosis [43].

In conclusion, the present study revealed that S-MPESCC was significantly different from SESCC in terms of clinicopathological characteristics include alcohol intake and pattern of LNM. The lymphatic flow is proved to be more complex and more prone to occur especially in the upper mediastinal and abdominal regions for patients with S-MPESCC. In addition, the OS was not significantly different between patients with S-MPESCC and SESCC, but a subgroup analysis of patients in the T3-4 group showed worse results for S-MPESCC survival. However, the present study had several limitations. First, we selected data from our own institute, and a certain loss of follow-up as a retrospective analysis was present. Second, some cases with preoperative neoadjuvant therapy were removed to standardize the pattern of LNM of S-MPESCC. Thus, this study had small sample size and only included 50 target cases, which might lead to a slight deficiency in the analyses of LNM and survival prognosis. Further comparative study still needs to be done by expanding the sample size of S-MPESCC.

## Supplementary Information

**Additional file 1:** **Supplementary Table 1. **Different tumor location groups in 50 cases of S-MPESCC including primary cancer and multiple cancer.

**Additional file 2:** **Supplementary Table 2.** The depth of tumor between the primary and multiple lesions.

**Additional file 3:** **Supplementary Fig. 1. **The receiver operating characteristic (ROC) curve showing the cutoff of relevant variables, including length of primary cancer (AUC = 0.638, SE =0.082, P = 0.105, 95%CI = 0.476-0.799) and dissection number of lymph nodes(AUC = 0.588, SE = 0.083, P = 0.299, 95%CI = 0.426-0.751).

## Data Availability

The datasets used and/or analyzed during the current study are available from the corresponding author on reasonable request.

## Abbreviations

S-MPESCC: Synchronous multiple primary esophageal squamous cell carcinoma
SESCC: Single esophageal squamous cell carcinoma
MPC: Multiple primary carcinoma
MPWEC: MPC with esophageal cancer
SMPWEC: Synchronous MPWEC
LNM: Lymph node metastasis
LNs: Lymph nodes
LNR: Lymph node rate
OS: Overall survival
ROC: Receiver operating characteristic
HR: Hazard ratio
CI: Confidence interval
EC: Esophageal cancer
ALDH2: Alcoholics with inactive aldehyde dehydrogenase-2
NBI: Narrow band imaging
ESD: Endoscopic submucosal dissection
NCCN: National Comprehensive Cancer Network
