# Supplementary material for: Comparison of clinicopathological features and prognostic significance between synchronous multiple primary and solitary esophageal squamous cell carcinomas
Source: BMC Cancer. 2022 Nov 19;22:1191. doi: 10.1186/s12885-022-10283-2 (PMC9675276; doi:10.1186/s12885-022-10283-2)
Supplement: Supplementary file 1 — Additional file 1: Supplementary Table 1. Different tumor location groups in 50 cases of S-MPESCC including primary cancer and multiple cancer. [file 12885_2022_10283_MOESM1_ESM.docx]

**Supplementary Table 1** Different tumor location groups in 50 cases of S-MPESCC including primary cancer and multiple cancer

| Group | primary cancer | multiple cancer | Total |
| --- | --- | --- | --- |
| U-m | Upper | middle | 2(4.0%) |
| U-l | Upper | lower | 1(2.0%) |
| M-u | Middle | upper | 3(6.0%) |
| M-m | Middle | middle | 16(32.0%) |
| M-l | Middle | lower | 13(26.0%) |
| L-u | Lower | upper | 1(2.0%) |
| L-m | Lower | middle | 12(24.0%) |
| L-l | Lower | lower | 2(4.0%) |

*S-MPESCC synchronous multiple primary esophageal squamous cell carcinoma*
