# Supplementary material for: Comparison of clinicopathological features and prognostic significance between synchronous multiple primary and solitary esophageal squamous cell carcinomas
Source: BMC Cancer. 2022 Nov 19;22:1191. doi: 10.1186/s12885-022-10283-2 (PMC9675276; doi:10.1186/s12885-022-10283-2)
Supplement: Supplementary file 2 — Additional file 2: Supplementary Table 2. The depth of tumor between the primary and multiple lesions. [file 12885_2022_10283_MOESM2_ESM.docx]

**Supplementary Table 2** The depth of tumor between the primary and multiple lesions

| Case | pT stage of primary cancer | pT stage of second cancer | pT stage of triple cancer |
| --- | --- | --- | --- |
| 1 | 1a | Tis |  |
| 2 | 1a | Tis |  |
| 3 | 1a | Tis |  |
| 4 | 1a | 1a |  |
| 5 | 1b | Tis |  |
| 6 | 1b | Tis |  |
| 7 | 1b | Tis |  |
| 8 | 1b | Tis |  |
| 9 | 1b | 1b |  |
| 10 | 1b | 1b |  |
| 11 | 1b | 1b |  |
| 12 | 1b | 1b | 1b |
| 13 | 2 | Tis |  |
| 14 | 2 | Tis |  |
| 15 | 2 | Tis |  |
| 16 | 2 | Tis |  |
| 17 | 2 | 1b |  |
| 18 | 2 | 1b |  |
| 19 | 2 | 2 |  |
| 20 | 2 | 2 |  |
| 21 | 2 | 2 |  |
| 22 | 3 | Tis |  |
| 23 | 3 | 1b |  |
| 24 | 3 | 1b |  |
| 25 | 3 | 1b |  |
| 26 | 3 | 1b |  |
| 27 | 3 | 1b |  |
| 28 | 3 | 2 |  |
| 29 | 3 | 3 |  |
| 30 | 3 | 3 |  |
| 31 | 3 | 3 |  |
| 32 | 3 | 3 |  |
| 33 | 3 | 3 |  |
| 34 | 3 | 3 |  |
| 35 | 3 | 3 | 3 |
| 36 | 3 | 3 | 3 |
| 37 | 4a | Tis |  |
| 38 | 4a | 1b |  |
| 39 | 4a | 1b |  |
| 40 | 4a | 1b | 1b |
| 41 | 4a | 1b | 1b |
| 42 | 4a | 2 | 2 |
| 43 | 4a | 4a |  |
| 44 | 4a | 4a |  |
| 45 | 4a | 4a |  |
| 46 | 4a | 4a |  |
| 47 | 4a | 4a |  |
| 48 | 4a | 4a |  |
| 49 | 4a | 4a |  |
| 50 | 4a | 4a |  |
