# Supplementary material for: Comparison of clinicopathological features and prognostic significance between synchronous multiple primary and solitary esophageal squamous cell carcinomas
Source: BMC Cancer. 2022 Nov 19;22:1191. doi: 10.1186/s12885-022-10283-2 (PMC9675276; doi:10.1186/s12885-022-10283-2)
Supplement: Supplementary file 3 — Additional file 3: Supplementary Fig. 1. The receiver operating characteristic (ROC) curve showing the cutoff of relevant variables, including length of primary cancer (AUC = 0.638, SE =0.082, P = 0.105, 95%CI = 0.476-0.799) and dissection number of lymph nodes(AUC = 0.588, SE = 0.083, P = 0.299, 95%CI = 0.426-0.751). [file 12885_2022_10283_MOESM3_ESM.docx]

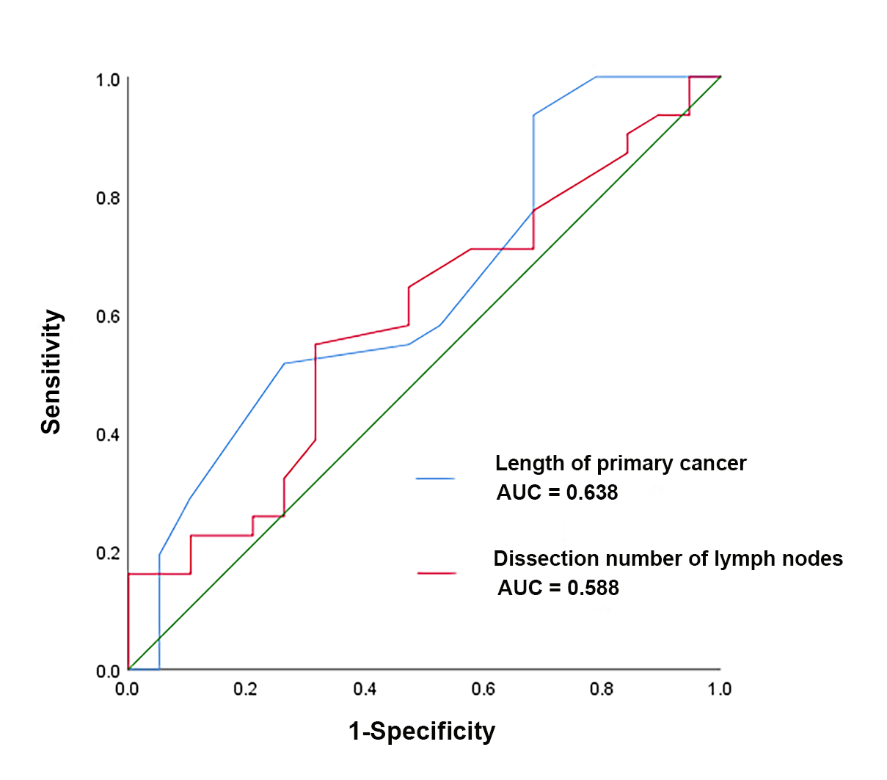


**Supplementary Fig. 1** The receiver operating characteristic (ROC) curve showing the cutoff of relevant variables, including length of primary cancer (AUC = 0.638, SE = 0.082, P = 0.105, 95%CI = 0.476-0.799) and dissection number of lymph nodes (AUC = 0.588, SE = 0.083, P = 0.299, 95%CI = 0.426-0.751).
